# Supplementary material for: Microbial adaptation to spaceflight is correlated with bacteriophage-encoded functions
Source: Nat Commun. 2024 May 15;15:3474. doi: 10.1038/s41467-023-42104-w (PMC11096397; doi:10.1038/s41467-023-42104-w)
Supplement: Supplementary file 1 — Supplementary Information [file 41467_2023_42104_MOESM1_ESM.pdf]

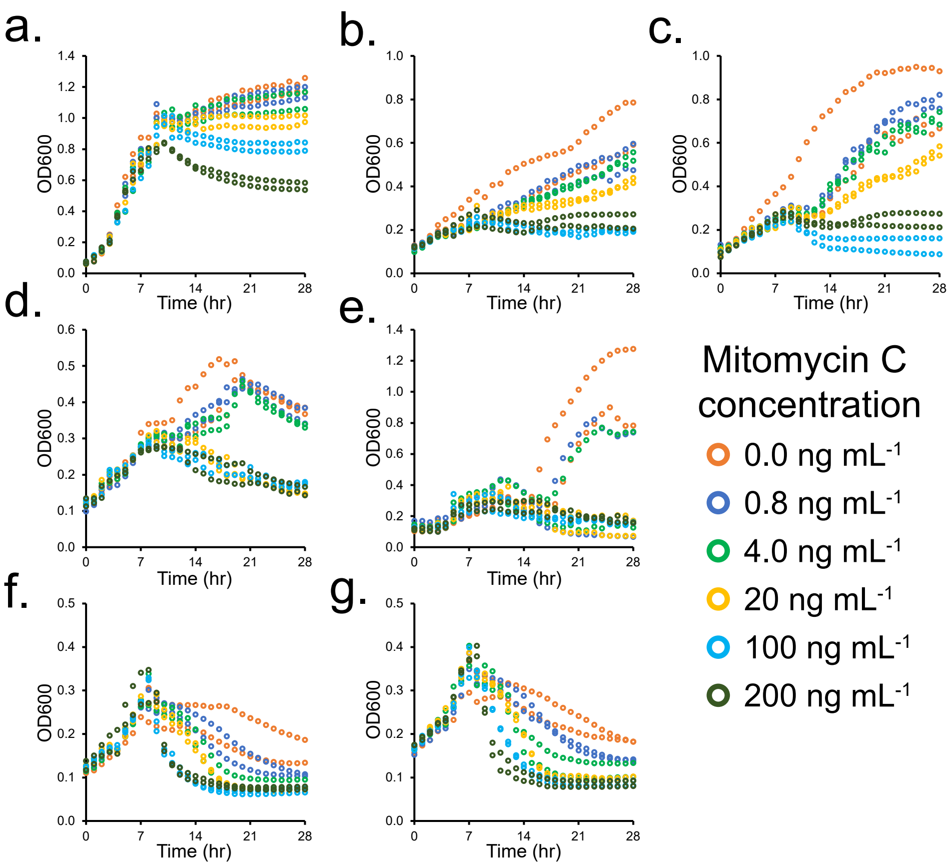

Supplementary Figure 1: ISS isolates treated with increasing concentrations of mitomycin C. a) *Pseudomonas fulva* F8_7S_9B, b) *Staphylococcus saprophyticus* F6_7S_P5, c) *Staphylococcus saprophyticus* F6_7S_P13, d) *Bacillus amyloliquifaeciens* IIF7SW-P4, e) *Bacillus amyloliquifaeciens* IIF7SC-B1, f) *Paenibacillus polymyxa* IIF5SW-B4, g) *Paenibacillus polymyxa* IIF8SW-P3.

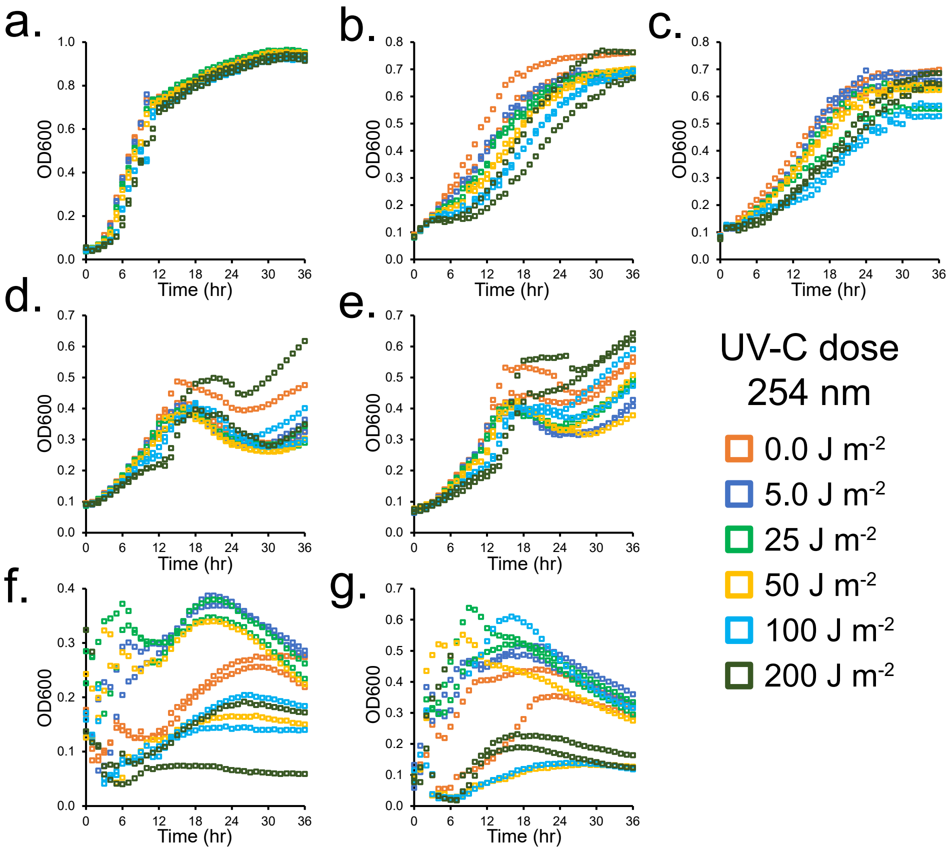

Supplementary Figure 2: ISS isolates treated with increasing concentrations of UV-C radiation (254 nm). a) *Pseudomonas fulva* F8_7S_9B, b) *Staphylococcus saprophyticus* F6_7S_P5, c) *Staphylococcus saprophyticus* F6_7S_P13, d) *Bacillus amyloliquifaeciens* IIF7SW-P4, e) *Bacillus amyloliquifaeciens* IIF7SC-B1, f) *Paenibacillus polymyxa* IIF5SW-B4, g) *Paenibacillus polymyxa* IIF8SW-P3.
