# Supplementary material for: Microbial adaptation to spaceflight is correlated with bacteriophage-encoded functions
Source: Nat Commun. 2024 May 15;15:3474. doi: 10.1038/s41467-023-42104-w (PMC11096397; doi:10.1038/s41467-023-42104-w)
Supplement: Supplementary file 3 — Reporting Summary [file 41467_2023_42104_MOESM3_ESM.pdf]

nature portfolio

Corresponding author(s): Jared T. Broddrick

Last updated by author(s): Sep 5, 2023

Reporting Summary

Nature Portfolio wishes to improve the reproducibility of the work that we publish. This form provides structure for consistency and transparency in reporting. For further information on Nature Portfolio policies, see our Editorial Policies and the Editorial Policy Checklist.

Statistics

For all statistical analyses, confirm that the following items are present in the figure legend, table legend, main text, or Methods section.

n/a	Confirmed	
☐	☒	The exact sample size (*n*) for each experimental group/condition, given as a discrete number and unit of measurement
☒	☐	A statement on whether measurements were taken from distinct samples or whether the same sample was measured repeatedly
☐	☒	The statistical test(s) used AND whether they are one- or two-sided<br>*Only common tests should be described solely by name; describe more complex techniques in the Methods section.*
☒	☐	A description of all covariates tested
☐	☒	A description of any assumptions or corrections, such as tests of normality and adjustment for multiple comparisons
☐	☒	A full description of the statistical parameters including central tendency (e.g. means) or other basic estimates (e.g. regression coefficient) AND variation (e.g. standard deviation) or associated estimates of uncertainty (e.g. confidence intervals)
☐	☒	For null hypothesis testing, the test statistic (e.g. *F*, *t*, *r*) with confidence intervals, effect sizes, degrees of freedom and *P* value noted<br>*Give P values as exact values whenever suitable.*
☒	☐	For Bayesian analysis, information on the choice of priors and Markov chain Monte Carlo settings
☒	☐	For hierarchical and complex designs, identification of the appropriate level for tests and full reporting of outcomes
☒	☐	Estimates of effect sizes (e.g. Cohen's *d*, Pearson's *r*), indicating how they were calculated

Our web collection on statistics for biologists contains articles on many of the points above.

Software and code

Policy information about availability of computer code

Data collection	NCBI web-based access, Oxford Nanopore Tech Guppy basecaller (v6.2.1), Flye (v2.9.1), Oxford Nanopore Tech Medaka (v1.7.0).
Data analysis	PHASTER web-based interface, PhiSpy (v4.2.20), MeShClust (v3.0), NCBI nucleotide BLAST (web-server), anvi'o (v7.1), NCBI ORFinder (web-server), MicrobeAnnotator (v2.0.5), Python (v3.9), Jupyter Notebook (v1.0.0), Matplotlib (3.5.1), Seaborn (0.11.2), Numpy (1.22.3), Pandas (1.4.2), Biopython (1.79), SciPy (1.7.3), ABRicate (v1.0.0), IMG/VR (v4, accessed July 2023), Virulence Factor Database (accessed April 2023).

For manuscripts utilizing custom algorithms or software that are central to the research but not yet described in published literature, software must be made available to editors and reviewers. We strongly encourage code deposition in a community repository (e.g. GitHub). See the Nature Portfolio guidelines for submitting code & software for further information.

Data

Policy information about availability of data

All manuscripts must include a data availability statement. This statement should provide the following information, where applicable:

- Accession codes, unique identifiers, or web links for publicly available datasets
- A description of any restrictions on data availability
- For clinical datasets or third party data, please ensure that the statement adheres to our policy

The long-read assemblies and raw fast5 files generated in this study have been deposited in the NASA Open Science Data Repository (https://osdr.nasa.gov/) under accession code OSD-582 (https://osdr.nasa.gov/bio/repo/data/studies/OSD-582, DOI: 10.26030/v6s6-w170) and in the NCBI databases Assembly under accession

codes GCF_017167745.1 (https://www.ncbi.nlm.nih.gov/datasets/genome/GCF_017167745.1) and GCF_017166245.1 (https://www.ncbi.nlm.nih.gov/datasets/genome/GCF_017166245.1), BioProject under accession code PRJNA994947 (https://www.ncbi.nlm.nih.gov/bioproject/?term=PRJNA994947), BioSamples under accession codes SAMN36453224 (https://www.ncbi.nlm.nih.gov/biosample/36453225) and SAMN36453224 (https://www.ncbi.nlm.nih.gov/biosample/36453224), and SRA under accession codes SRR25281509 (https://www.ncbi.nlm.nih.gov/sra/SRX21026417[accn]) and SRR25281510 (https://www.ncbi.nlm.nih.gov/sra/SRX21026416[accn]). All data for figures have been uploaded to Figshare (DOI: 10.6084/m9.figshare.23290226). Source data are provided with this paper.

Human research participants

Policy information about studies involving human research participants and Sex and Gender in Research.

Reporting on sex and gender	N/A
Population characteristics	N/A
Recruitment	N/A
Ethics oversight	N/A

Note that full information on the approval of the study protocol must also be provided in the manuscript.

Field-specific reporting

Please select the one below that is the best fit for your research. If you are not sure, read the appropriate sections before making your selection.

☒ Life sciences ☐ Behavioural & social sciences ☐ Ecological, evolutionary & environmental sciences

For a reference copy of the document with all sections, see nature.com/documents/nr-reporting-summary-flat.pdf

Life sciences study design

All studies must disclose on these points even when the disclosure is negative.

Sample size	Sample size was determined based on the number of available genomes for each species at the experimental conditions (spaceflight versus terrestrial).
Data exclusions	Species that had either spaceflight or terrestrial genome counts of less than 8 were excluded.
Replication	The work was analysis of existing available genomes in public databases and no replication was required. The output of data analysis was reproduced on four separate occasions on both authors' respective compute solutions and yielded the same outputs.
Randomization	Randomization was not performed as the entire population of available genomes were used (not down-selected) and the "treatment" variable used (spaceflight versus non-spaceflight) was not a treatment we could apply to the dataset, as it is inherent.
Blinding	Blinding was performed at the initial pangenome stage, as all genomes for a given species were included in the analysis. Binding was not performed on the subsequent analysis due to the fact that the treatment variable, spaceflight, was selected as the explanatory variable.

Reporting for specific materials, systems and methods

We require information from authors about some types of materials, experimental systems and methods used in many studies. Here, indicate whether each material, system or method listed is relevant to your study. If you are not sure if a list item applies to your research, read the appropriate section before selecting a response.

Materials & experimental systems

n/a	Involved in the study	
☒	☐	Antibodies
☒	☐	Eukaryotic cell lines
☒	☐	Palaeontology and archaeology
☒	☐	Animals and other organisms
☒	☐	Clinical data
☒	☐	Dual use research of concern

Methods

n/a	Involved in the study	
☒	☐	ChIP-seq
☒	☐	Flow cytometry
☒	☐	MRI-based neuroimaging
