# Supplementary material for: Microbial adaptation to spaceflight is correlated with bacteriophage-encoded functions
Source: Nat Commun. 2024 May 15;15:3474. doi: 10.1038/s41467-023-42104-w (PMC11096397; doi:10.1038/s41467-023-42104-w)
Supplement: Supplementary file 4 — Description of Additional Supplementary Files [file 41467_2023_42104_MOESM4_ESM.pdf]

Description of additional supplementary files

Title: Supplementary Data 1.

Description: Excel file containing tabs for 1) Genome information used in the study, 2) Genome comparison showing the similarity between flight isolates 3) Intact prophage clusters, 4) Long-read assembly prophage mapping, 5) Complete functional annotation of the prophage-encoded proteins, 6) Functional annotation of prophage-encoded proteins that are non-redundant with host genome functions, 7) AMR genes corroborated with ABRicate, 8) NCBI Accessions for Terrestrial genomes, 9) sequence information for all prophage regions.
